# Seven vs Fourteen Days of Antibiotics for Gram-Negative Bloodstream Infection: A Systematic Review and Noninferiority Meta-Analysis

**DOI:** 10.1001/jamanetworkopen.2025.1421

**Published:** 2025-03-21

**Authors:** Todd C. Lee, Connor J. Prosty, Michael Fralick, Angela Huttner, Emily G. McDonald, José Molina, Mical Paul, Ruxandra Pinto, Asgar Rishu, Elodie von Dach, Dafna Yahav, Rob Fowler, Nick Daneman

**Affiliations:** 1 Division of Infectious Diseases, Department of Medicine, McGill University, Montreal, Quebec, Canada; 2 Faculty of Medicine, McGill University, Montreal, Quebec, Canada; 3 Department of Medicine, Sinai Health and University of Toronto, Toronto, Ontario, Canada; 4 Division of Infectious Diseases, Department of Medicine, Geneva University Hospital, Geneva, Switzerland; 5 Division of General Internal Medicine, Department of Medicine, Research Institute of the McGill University Health Centre, Montreal, Quebec, Canada; 6 Unit of Infectious Diseases, Microbiology and Parasitology, Virgen del Rocío University Hospital, Seville, Spain; 7 Institute of Biomedicine of Seville (IBiS), Virgen del Rocío University Hospital/CSIC/University of Seville, Seville, Spain; 8 CIBER de Enfermedades Infecciosas, Instituto de Salud Carlos III, Madrid, Spain; 9 Infectious Diseases, Rambam Health Care Campus, Technion–Israel Institute of Technology, Haifa, Israel; 10 Department of Critical Care Medicine, Sunnybrook Health Sciences Center, University of Toronto, Institute of Health Policy, Management, and Evaluation, Toronto, Ontario, Canada; 11 Sunnybrook Research Institute, Sunnybrook Health Sciences Centre, University of Toronto, Toronto, Ontario, Canada; 12 Clinical Research Center, Geneva University Hospitals, Geneva, Switzerland; 13 Infectious Diseases Unit, Sheba Medical Center, Faculty of Medical & Health Sciences, Tel Aviv University, Ramat-Gan, Israel; 14 Department of Medicine, University of Toronto, Toronto, Ontario, Canada; 15 Division of Infectious Diseases, Department of Medicine, Sunnybrook Health Sciences Centre, University of Toronto, Toronto, Ontario, Canada

## Abstract

**Importance:**

Gram-negative bloodstream infections are a common cause of hospitalization. A 2-week duration of antibiotic therapy has been commonly used, but shorter durations may have similar outcomes.

**Objectives:**

To assess whether 7 days of antibiotic therapy was noninferior to 14 days.

**Data Sources:**

Starting with a 2022 individual patient data meta-analysis, PubMed, Cochrane Central Register of Controlled Trials, and Web of Science were searched to identify additional eligible randomized clinical trials (RCTs) conducted from May 1, 2022, until November 30, 2024.

**Study Selection:**

RCTs involving primarily adults who were hospitalized at the time of Gram-negative bloodstream infection and were allocated to 7 or 14 days of antibiotic therapy. Studies were independently reviewed by 2 investigators.

**Data Extraction and Synthesis:**

PRISMA guidelines were followed. Data were extracted by 2 investigators. Any unpublished data were obtained directly from study authors. Risk of bias and certainty of the evidence were assessed in duplicate using the Cochrane Risk of Bias Tool, version 2, and the Grading of Recommendations Assessment, Development and Evaluation approach. Data were pooled by separate random-effects meta-analyses for the intention-to-treat (ITT) and per-protocol (PP) populations. A noninformative prior probability was used for the effect, and an evidence-based weakly informative prior probability was used for heterogeneity. Risk ratios (RRs), 95% credible intervals (CrIs), and probability of noninferiority were calculated using a prespecified upper bound of 1.25 or less.

**Main Outcomes and Measures:**

Ninety-day all-cause mortality.

**Results:**

Four eligible RCTs contributed 3729 patients in the ITT population (1912 women [51.3%]; median age range, 67-79 years) and 3126 in the PP population. In the ITT analysis, within 90 days, 226 patients (12.0%) receiving 7 days of antibiotics died compared with 253 (13.7%) receiving 14 days, corresponding to an RR for 90-day mortality of 0.91 (95% CrI, 0.69-1.22) and a 97.8% probability of noninferiority. In the PP analysis, the RR was 0.93 (95% CrI, 0.68-1.32), corresponding to a 95.1% probability of noninferiority.

**Conclusions and Relevance:**

In this systematic review and meta-analysis of patients with Gram-negative bloodstream infections and adequate source control, 7 days of antibiotic therapy had a high probability of being noninferior to 14 days. These findings support a shorter duration of antibiotic therapy for appropriately selected patients like those in the included RCTs.

## Key Points

**Question:**

Are 7 days of antibiotic therapy adequate for the treatment of most Gram-negative bloodstream infections?

**Findings:**

In this systematic review and meta-analysis of 4 randomized clinical trials including 3729 patients, 7 days of antibiotics had a 97.8% probability of being noninferior to 14 days for the outcome of 90-day mortality.

**Meaning:**

These findings suggest that most Gram-negative bloodstream infections can be treated with 7 days of antibiotics unless there is a concern for inadequate source control.

## Teaser

This systematic review and noninferiority meta-analysis applies bayesian statistics to randomized clinical trials comparing 7 vs 14 days of antibiotic therapy among patients with Gram-negative bloodstream infections.

## Introduction

The optimal duration of antibiotic therapy for Gram-negative bloodstream infections is uncertain. While therapy has commonly been given for 2 weeks,^1,2^ recent randomized clinical trials (RCTs) have evaluated the noninferiority of shorter courses (eg, 7 days)^3,4^ and/or biomarker-directed therapy,^5^ compared with a longer duration (eg, 14 days). These 3 RCTs^3,4,5^ informed an individual patient data meta-analysis in 2023 (N = 1186).^6^ Since then, the Bacteremia Antibiotic Length Actually Needed for Clinical Effectiveness (BALANCE) trial was published,^7^ powered on an overall noninferiority margin of 4% for 90-day mortality; the study included 2547 patients with Gram-negative bloodstream infections. Within the subgroup of patients with Gram-negative bloodstream infection, 7 days of therapy were noninferior to 14 days in the intention-to-treat (ITT) analysis (risk difference, −2.8%; 95% CI, −5.6% to −0.1%).

Systematic review with meta-analysis can provide more accurate results on the efficacy of a medical intervention by combining data from individual studies. However, even the meta-analysis of data from multiple RCTs can fail to identify a superior therapy when the CIs of the pooled estimate cross the null. This is particularly true for meta-analyses containing noninferiority trials.^8^ Reporting summary results from meta-analyses of noninferiority trials that are based on a dichotomized interpretation of a *P* value of less than .05 ultimately fails to account for the statistical design of noninferiority studies, which are not expected to show a significant difference. Instead, a priori noninferiority margins can be selected and bayesian statistics used to provide a more valid estimate of the probability of noninferiority across different margins. The objective of this systematic review and meta-analysis was to apply this technique to RCTs comparing 7 vs 14 days of therapy for Gram-negative bloodstream infections.

## Methods

### Information Sources and Search Strategies

The protocol for this systematic review and meta-analysis was prespecified and is available on the Open Science Framework.^9^ The reporting follows the Preferred Reporting Items for Systematic Reviews and Meta-Analyses (PRISMA) framework. Starting with the existing individual patient data meta-analysis,^6^ we searched PubMed, Cochrane Central Register of Controlled Trials, and Web of Science to identify additional eligible RCTs conducted from May 1, 2022, until November 30, 2024. The search strategy combined the Cochrane highly sensitive filter for RCTs^10^ with the terms *duration* OR *days* and *antibiotic* and *bloodstream* OR *bacteremia* OR *bacteraemia*. We applied no language or publication restrictions. References of all included trials were hand searched for additional relevant trials.

Participants were adults hospitalized with Gram-negative bloodstream infection and allocated to 7 or 14 days of therapy. Newly identified studies were combined with those previously reported in the individual patient data meta-analysis. The primary outcome was 90-day all-cause mortality as evaluated by both intention-to-treat (ITT) and per-protocol (PP) analyses.

### Data Collection and Analysis

Studies were independently reviewed and appraised by 2 reviewers, with disagreement resolved by consensus with a third reviewer. Where data were not available in any manuscript, they were obtained directly from the study authors. Risk of bias was assessed using the Cochrane Risk of Bias Tool, version 2,^11^ by 2 independent reviewers (T.C.L. and C.J.P.) and visualized using the risk-of-bias visualization (robvis).^12^ We do not report participant race and ethnicity because it was not reported in the Table 1 of 3 of the 4 included trials.

### Certainty of the Evidence

The certainty of evidence for the primary and secondary outcomes was evaluated in duplicate by independent reviewers using the Grading of Recommendations Assessment, Development and Evaluation (GRADE) criteria.^13^ Findings were presented using the GRADEpro guideline development tool.^14^

### Statistical Analysis

Differences in 90-day mortality in patients with Gram-negative bloodstream infections were compared between 7 and 14 days of antibiotic therapy. We conducted a bayesian meta-analysis using the bayesmeta module^15^ in R, version 4.1.3 (R Foundation for Statistical Computing). We used a noninformative prior probability (N ~ [0,10^2^]) for the effect (μ statistic). Heterogeneity was quantified using the parameter (τ statistic), and we used a weakly informative prior based on a collection of systematic reviews comparing pharmacological interventions with controls and reporting mortality outcomes.^16^ We conducted the meta-analysis on the log risk ratio (RR) scale for both the ITT and PP populations and report the results exponentiated with 95% credible intervals (CrIs). Noninferiority was defined a priori as an RR of 1.25 or less because the control event rate was anticipated to differ across trials, and a larger absolute noninferiority margin might not be appropriate at lower event rates. An RR of 1.25 or less corresponds to an absolute noninferiority margin of 2.5% at 10% control event rates, 3.75% at 15% control event rates, and 5% at 20% control event rates. We further report the probability of noninferiority (RR ≤1.25) and superiority (RR <1.00). Finally, recognizing that there is no consensus on the optimal noninferiority margin, we generated a graph of the probability of noninferiority (or superiority) across the range of RRs between 0.70 and 1.30. In a prior systematic review on the relative sizes of noninferiority margins in RCTs,^17^ the median relative noninferiority margin for mortality end points was an RR of 1.3 (95% CI, 1.2-1.6), which aligns with the margin we selected.

## Results

**Figure 1.**
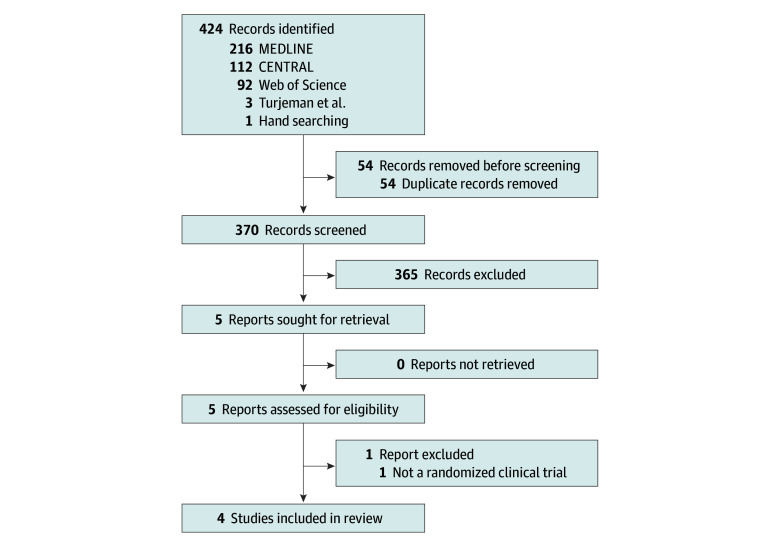
PRISMA Diagram PRISMA indicates Preferred Reporting Items for Systematic Reviews and Meta-Analyses.

The study flowchart is presented in Figure 1. Four trials met our inclusion criteria. Three of these^3,4,5^ were previously combined in an individual patient meta-analysis^6^; the fourth RCT was the BALANCE trial.^7^ Trial characteristics are described in Table 1 and patient characteristics in Table 2. A total of 3729 participants were included in the ITT analysis (1817 [48.7%] men and 1912 [51.3%] women; median age range, 67-79 years) and 3126 in the PP analysis. Most infections were caused by species in the Enterobacterales order (3385 of 3729 [90.8%]), with one trial focused only on Enterobacterales bloodstream infection^3^ and the others allowing other Gram-negative bacteria.^4,5,7^ In all trials, the choice and route of administration of antibiotics was decided by the treating team, and treatment duration was unmasked, either at the time of randomization or at day 7. As open-label trials, all had some concern for bias due to deviation from the intended intervention but were otherwise considered at low risk of bias (eFigure in Supplement 1).

**Table 1.** Characteristics of the Included Studies

Source	Location	Blinding	Inclusion criteria	Exclusion criteria	Timing of randomization
Yahav et al,^4^ 2019	Israel and Italy	Open-label	Bacteremia caused by aerobic Gram-negative pathogen, afebrile, and hemodynamically stable for ≥48 h at d 7	Sources of bacteremia other than urinary, intra-abdominal, respiratory, central venous catheter, skin or soft tissue, or unknown; source of infection uncontrolled, polymicrobial, *Salmonella* or *Brucella* infection, or immunosuppression from neutropenia, HIV, or recent allogeneic stem cell transplant	48 h Without fever and with hemodynamic stability to 7 d from the positive culture
von Dach et al,^5^ 2020	Switzerland	Open-label	Gram-negative bacteremia with growth in ≥1 blood culture and treatment with a microbiologically active antibiotic	Hemodynamic instability or fever <24 h preceding enrollment, severe immunosuppression, bacteremia that is polymicrobial or from nonfermenting bacilli, Gram-positive bacteremia, bacteremia <60 d preceding this episode, or complicated infections (ie, endocarditis or abscess)	Day 5 from the positive culture
Molina et al,^3^ 2022	Spain	Open-label	Adults with Enterobacterales bacteremia	Pregnancy, uncontrolled source and not expected to be controlled <24 h, undergoing chemotherapy and with neutropenia expected >7 d, sites of infection requiring longer courses of antibiotics (ie, osteomyelitis, meningitis, and prostatitis), concomitant infection unrelated to the bacteremia requiring treatment, infection with carbapenemase-producing organisms, polymicrobial bacteremia, or survival estimated <48 h	Identification of the pathogen <48 h
BALANCE Investigators,^7^ 2024	Canada, Australia, New Zealand, Saudi Arabia, Israel, Switzerland, US	Open-label	Hospitalized patients with blood culture containing a pathogen	Severe immune system compromise, defined by absolute neutrophil count <500 μ/L or receiving immunosuppressive treatment for solid organ or bone marrow or stem cell transplant; prosthetic heart valve or synthetic endovascular graft (post major vessel repair with synthetic material); documented or suspected syndrome with well-defined requirement for prolonged treatment (eg, endocarditis, osteomyelitis or septic arthritis, undrainable or undrained abscess, unremovable or unremoved prosthetic-associated infection); single positive blood culture with a common contaminant organism; *Staphylococcus aureus* or *lugdunensis*; *Candida* species or other fungal species	Adequate treatment v <7 d with allocation concealed until 7 d

SI conversion factor: To convert neutrophils to ×10^9^/L, multiply by 0.001.

**Table 2.** Patient Characteristics

Source	No. randomized (N = 3729)	ICU admission at study entry, No. (%)	Sex, No. (%)		Age, median (IQR), y	Urinary source, No. (%)	Enterobacterales infection, No. (%)
			Women	Men			
Yahav et al,^4^ 2019	604	0	319 (52.8)	285 (47.2)	71 (61-80)	411 (68.0)	543 (89.9)
von Dach et al,^5^ 2020	334^a^	0	201 (60.2)	133 (39.8)	79 (68-86)	224 (67.1)	334 (100)
Molina et al,^3^ 2022	248	21 (8.5)^b^^,^^c^	118 (47.6)	130 (52.4)	67 (53-77)	136 (54.8)	248 (100)
BALANCE Investigators,^7^ 2024	2547	1298 (51.0)	1276 (50.1)	1271 (49.9)	71.5 (60-81)	1347 (52.9)	2260 (88.7)

Abbreviation: ICU, intensive care unit.

^a^ One hundred and sixty-nine more patients were randomized to C-reactive protein–directed therapy.

^b^ Includes patients who were in the ICU within 30 days of randomization.

^c^ Ninety-day mortality data were available for 244 patients.

**Figure 2.**
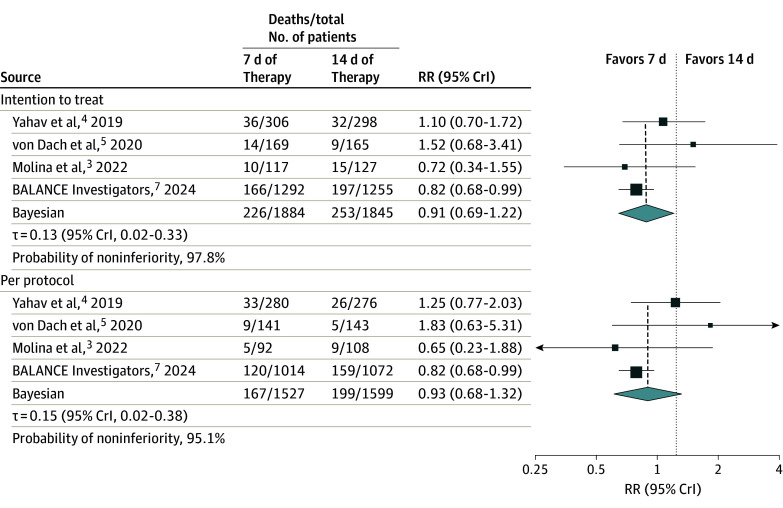
Forest Plot of Included Studies The top panel shows the intention-to-treat results and the lower panel the per-protocol results. The prespecified noninferiority margin (risk ratio [RR], 1.25) is indicated by the vertical dotted line. The vertical dashed lines show the point estimate for the pooled results. Size of squares indicates the relative weight of the individual study; diamonds, the pooled RR and 95% credible interval (CrI). BALANCE indicates Bacteremia Antibiotic Length Actually Needed for Clinical Effectiveness.

Overall, 1884 patients were assigned to the 7-day and 1845 to the 14-day arm. For the ITT analysis (Figure 2, top), the RR for mortality with 7 vs 14 days of therapy was 0.91 (95% CrI, 0.69-1.22), corresponding to a 97.8% probability of noninferiority. The control event rate was 13.7% (253 of 1845), implying the noninferiority margin would correspond to 3.4% on the absolute scale. For comparison, the overall experimental event rate was 12.0% (226 of 1884).

In the PP analysis, 1527 patients were assigned to 7 days and 1599 to 14 days (Figure 2, bottom). The RR for mortality with 7 vs 14 days of therapy was 0.93 (95% CrI, 0.68-1.32), which corresponded to a 95.1% probability of noninferiority. The control event rate was 12.4% (199 of 1599), corresponding to a noninferiority margin of 3.1% on the absolute scale.

**Figure 3.**
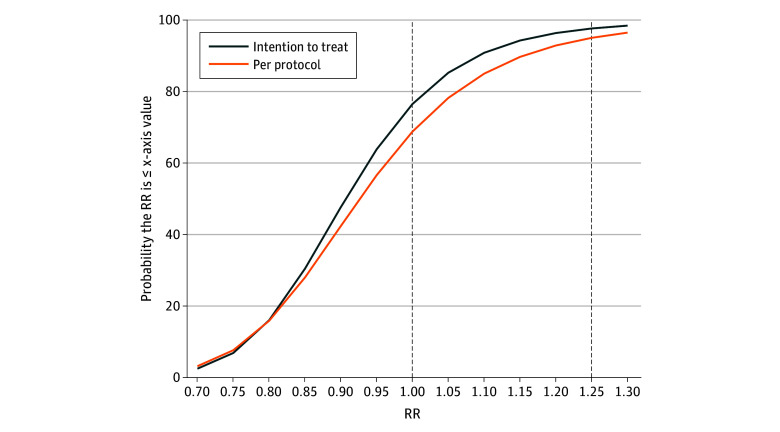
Probability of Noninferiority (or Superiority) as a Function of the Upper Bound The y-axis represents the probability of a result less than or equal to the x-axis value. For example, at the prespecified upper bound of 1.25, the probability that the risk ratio (RR) is 1.25 or less is 97.8% for the intention-to-treat analysis and 95.1% for the per-protocol analysis. The dashed line at 1.00 represents superiority (RR is ≤1.00); the dashed line at 1.25 represents noninferiority.

The probability of superiority (RR <1.00) was 76.6% in the ITT analysis and 68.9% in the PP analysis. The probability of noninferiority or superiority vs the upper limit of the RR is presented in Figure 3.

### Certainty of the Evidence

For a common clinical presentation, with 4 RCTs at no risk of serious bias, we graded the certainty of the evidence as high. We graded the importance of the outcome as critical (eTable in Supplement 1).

## Discussion

In this systematic review and meta-analysis of 4 RCTs with a total of 3729 patients, we found that 7 days of therapy had a 95.1% (PP) to 97.8% (ITT) probability of noninferiority to 14 days of therapy for Gram-negative bloodstream infection with adequate source control. We also provide an analysis that allows the reader to estimate the probability of noninferiority for a variety of margins. In contrast to the prior meta-analysis on this topic,^6^ we include the results of the BALANCE RCT^7^ and use a prespecified noninferiority margin to contextualize results that are not statistically significant for superiority. This outcome is expected when performing a meta-analysis of noninferiority trials, and the method we have used can simultaneously evaluate superiority or noninferiority or even extend to equivalence by prespecifying a lower bound for the 95% CrI.

### Limitations

Limitations of this meta-analysis include a conservative estimate of the upper bound of the 95% CrI, given there are only 4 RCTs. The smaller sample size in the PP group also leads to wider 95% CrIs and slightly less probability of noninferiority. Nonetheless, even in the PP group, the probability of noninferiority exceeded 95% with a 68.9% probability of superiority with shorter treatment duration. While mechanisms of potential superiority are speculative, it is plausible that longer durations of antibiotic therapy could increase iatrogenic morbidity and mortality, perhaps through longer durations of hospitalization, requirements for intravenous access, the promotion of antibiotic resistance or dysbiosis, or increasing the risk of antibiotic-related adverse events.^18^ Other limitations mainly stem from the limitations of the included studies. It should be noted that certain patient groups are underrepresented in the trials included (eg, those with immunocompromise, such as patients with solid-organ transplant), the predominant sources of infection were urinary, and most of the data pertain to Enterobacterales bacteria. Ongoing trials in bloodstream infections due to *Pseudomonas aeruginosa*^19^ will provide important complementary evidence outside the dominant pathogens. Similarly, personalized therapy based on clinical stopping rules or biomarker levels^5^ would benefit from additional evaluation.

## Conclusions

In this systematic review and meta-analysis, 7 days of therapy likely represent the preferred duration for uncomplicated Gram-negative bloodstream infections. Future RCTs outside Enterobacterales bacteria and in populations that are more severely immunocompromised will be helpful in providing further evidence in support of shorter durations.

## Notes

Supplement 1. **eFigure.** Risk of Bias using Cochrane Risk of Bias Tool, Version 2 **eTable.** GRADE Rating of the Certainty of Evidence That 7 Days is Noninferior to 14 Days of Antibiotics for Gram-Negative Bloodstream Infections

Supplement 2.
Data Sharing Statement
