# Error in Affiliations and Results

**DOI:** 10.1001/jamanetworkopen.2025.12343

**Published:** 2025-04-24

In the Original Investigation titled “Seven vs Fourteen Days of Antibiotics for Gram-Negative Bloodstream Infection: A Systematic Review and Noninferiority Meta-Analysis,”^1^ published March 21, 2025, the affiliations for author José Molina were incomplete; the full correct affiliations are the Unit of Infectious Diseases, Microbiology and Parasitology, Virgen del Rocío University Hospital, Seville, Spain; Institute of Biomedicine of Seville (IBiS), Virgen del Rocío University Hospital/CSIC/University of Seville, Seville, Spain; and CIBER de Enfermedades Infecciosas, Instituto de Salud Carlos III, Madrid, Spain. Additionally, in the abstract, Results, and the eTable in Supplement 1, the 90-day mortality rate for participants who received 7 days of antibiotics was incorrectly reported as 12.8%; the correct rate is 12.0%. This article has been corrected.^1^
