# Supplementary material for: Seven vs Fourteen Days of Antibiotics for Gram-Negative Bloodstream Infection: A Systematic Review and Noninferiority Meta-Analysis
Source: JAMA Netw Open. 2025 Mar 21;8(3):e251421. doi: 10.1001/jamanetworkopen.2025.1421 (PMC11929019; doi:10.1001/jamanetworkopen.2025.1421)
Supplement: Supplement 1. — eFigure. Risk of Bias using Cochrane Risk of Bias Tool, Version 2 eTable. GRADE Rating of the Certainty of Evidence That 7 Days is Noninferior to 14 Days of Antibiotics for Gram-Negative Bloodstream Infections [file jamanetwopen-e251421-s001.pdf]

Supplementary Online Content

Lee TC, Prosty CJ, Fralick M, et al. Seven vs fourteen days of antibiotics for Gram-negative bloodstream infection: a systematic review and noninferiority meta-analysis. *JAMA Netw Open.* 2025;8(3):e251421. doi:10.1001/jamanetworkopen.2025.1421

eFigure. Risk of Bias using Cochrane Risk of Bias Tool, Version 2

eTable. GRADE Rating of the Certainty of Evidence That 7 Days is Noninferior to 14 Days of Antibiotics for Gram-Negative Bloodstream Infections

This supplementary material has been provided by the authors to give readers additional information about their work.

© 2025 Lee TC et al. *JAMA Network Open*.

eFigure. Risk of Bias using Cochrane Risk of Bias Tool, Version 2

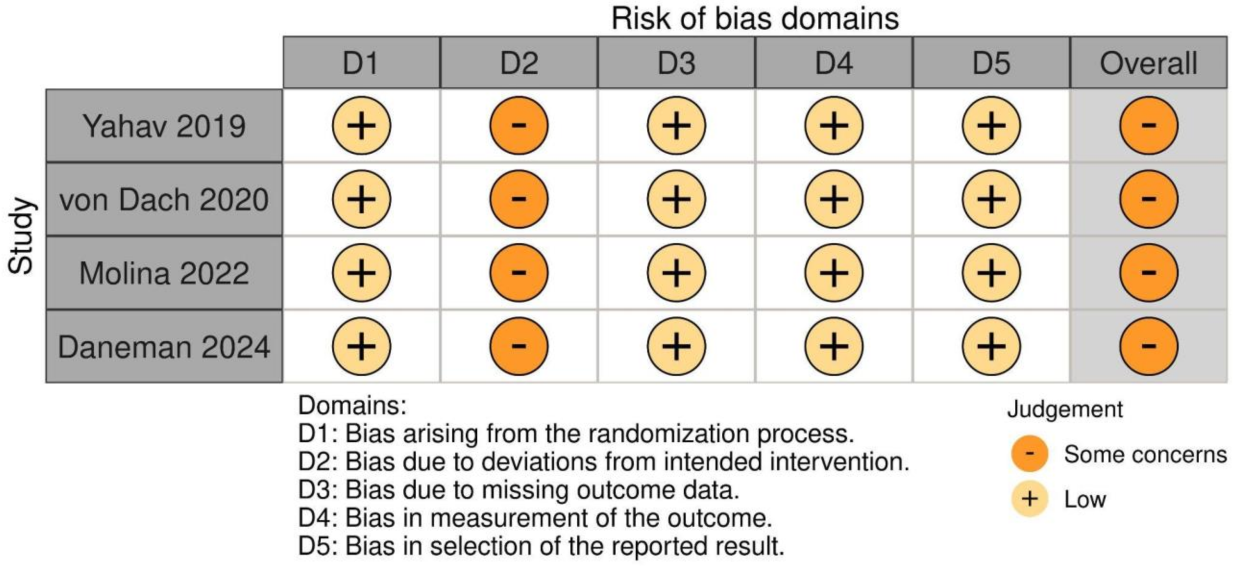

© 2025 Lee TC et al. *JAMA Network Open*.

eTable. GRADE Rating of the Certainty of Evidence That 7 Days is Noninferior to 14 Days of Antibiotics for Gram-Negative Bloodstream Infections

Certainty assessment							№ of patients		Effect		Certainty	Importance
№ of studies	Study design	Risk of bias	Inconsistency	Indirectness	Imprecision	Other considerations	7 days	14 days	Relative (95% CI)	Absolute (95% CI)		
**90-Day Mortality**												
4	Randomized trials	Not serious	Not serious	Not serious	Not serious	None	226/1884 (12.0%)	253/1845 (13.7%)	RR 0.91 (0.69 to 1.22)	12 fewer per 1,000 (from 43 fewer to 30 more)	⨁⨁⨁⨁ High	CRITICAL

CI: confidence interval; RR: risk ratio

© 2025 Lee TC et al. *JAMA Network Open*.
