# Supplementary material for: Seven vs Fourteen Days of Antibiotics for Gram-Negative Bloodstream Infection: A Systematic Review and Noninferiority Meta-Analysis
Source: JAMA Netw Open. 2025 Mar 21;8(3):e251421. doi: 10.1001/jamanetworkopen.2025.1421 (PMC11929019; doi:10.1001/jamanetworkopen.2025.1421)
Supplement: Supplement 2. — Data Sharing Statement [file jamanetwopen-e251421-s002.pdf]

Data Sharing Statement

Lee. Seven vs Fourteen Days of Antibiotics for Gram-Negative Bloodstream Infection. *JAMA Netw Open*. Published March 21, 2025. doi:10.1001/jamanetworkopen.2025.1421

Data
Data available: No

Additional Information
Explanation for why data not available: All data needed to re-create the analysis is in the paper.
